# Case Report: Rare case of signet ring gastric adenocarcinoma with rectal metastasis

**DOI:** 10.3389/fonc.2025.1573165

**Published:** 2025-07-31

**Authors:** Wan Izzah Wan Jaffar, Guo Hou Loo, Fazarina Mohammed, Deborah Chia Hsin Chew, Guhan Muthkumaran, Nik Ritza Kosai

**Affiliations:** ^1^ Department of General Surgery, Faculty of Medicine, National University of Malaysia, Kuala Lumpur, Malaysia; ^2^ Upper GI and Metabolic Surgery Unit, Faculty of Medicine, National University of Malaysia, Kuala Lumpur, Malaysia; ^3^ Department of Laboratory Diagnostic Services, Faculty of Medicine, National University of Malaysia, Kuala Lumpur, Malaysia; ^4^ Department of Gastroenterology and Hepatology, Faculty of Medicine, National University of Malaysia, Kuala Lumpur, Malaysia; ^5^ Department of Surgery, Faculty of Medicine, National University of Malaysia, Kuala Lumpur, Malaysia

**Keywords:** gastric cancer, signet-ring cell, rectal metastasis, peritoneal dissemination, palliative care

## Abstract

Gastric cancer remains a leading cause of cancer-related mortality worldwide, with the liver, peritoneum, lungs, and bones being the most common sites of metastasis. Rectal metastasis, also referred to as Schnitzler’s metastasis, is extremely rare and may clinically mimic primary rectal carcinoma, complicating diagnosis and delaying appropriate management. We report a case of a 69-year-old male diagnosed with signet-ring cell gastric adenocarcinoma who presented with symptoms of large bowel obstruction and constitutional decline. Cross-sectional imaging revealed a circumferential rectal mass and gastric wall thickening. Endoscopic biopsies of both gastric and rectal lesions confirmed poorly cohesive adenocarcinoma with signet ring features. Immunohistochemistry supported gastric origin of the rectal tumor. The patient underwent laparoscopic-assisted transverse colostomy for symptomatic relief but the planned gastrojejunostomy was abandoned due to extensive peritoneal involvement. He was treated with palliative chemotherapy (FOLFOX followed by modified de Gramont), achieving only transient radiologic response before clinical deterioration. Subsequent progression to lymphangitic carcinomatosis led to a shift toward best supportive care. This case highlights the diagnostic challenge posed by rectal metastasis from gastric cancer and underscores the aggressive nature and poor chemotherapy responsiveness of signet ring cell carcinoma. Clinicians should maintain high suspicion for secondary rectal lesions in patients with diffuse-type gastric cancer and lower gastrointestinal symptoms. Timely endoscopy, comprehensive histopathologic evaluation, and tailored palliative strategies remain essential to optimizing outcomes in such rare and complex presentations.

## Introduction

Gastric cancer (GC) is a significant global health burden, ranking as the fifth most commonly diagnosed malignancy and the fourth leading cause of cancer-related mortality worldwide as of 2020 (1). An estimated 1 million new cases and over 768,000 deaths were reported globally in that year alone, reflecting the disease’s aggressive nature and frequent late-stage diagnosis (1). The incidence of GC is particularly high in East Asia, including countries such as Japan, South Korea, and China, where both mortality rates and five-year prevalence rates exceed global averages (2). These regional disparities are partly attributed to dietary, genetic, and microbial risk factors, and are compounded by differences in national screening programs.

In Malaysia, gastric cancer is relatively less common but remains clinically significant, ranking as the tenth most frequent malignancy according to the Malaysia National Cancer Registry (2007–2011) (2). Notably, the proportion of advanced and metastatic presentations has been increasing over the past two decades, with current data suggesting that up to 40% of Malaysian GC cases are metastatic at diagnosis (3). This trend underscores the ongoing challenges in early detection and public health screening strategies.

The European Society for Medical Oncology (ESMO) has highlighted several demographic and etiological patterns: GC is twice as common in men as in women and tends to present in individuals aged over 50 (4). Anatomically, GC is classified into cardia and non-cardia subtypes. Non-cardia GC, which comprises approximately 80% of global cases, is more prevalent in East Asia and Latin America and is closely linked to *Helicobacter pylori* infection, high dietary salt, alcohol consumption, and low intake of fruits and vegetables (4). In contrast, cardia GC is more common in North America and Western Europe and is associated with obesity and chronic gastroesophageal reflux disease (GERD) (4).

From a molecular standpoint, GC is a heterogeneous disease. EBV-associated GC, for instance, is found in about 9% of cases globally and is more likely to localize to the fundus or body of the stomach. EBV-positive tumors typically exhibit dense lymphoid infiltration and may carry prognostic and therapeutic implications (4). Additionally, genomic profiling has identified distinct subtypes of GC, including microsatellite instability-high (MSI-H), chromosomal instability, and genomically stable tumors, the latter often encompassing diffuse and signet ring cell variants.

Despite advances in endoscopic and systemic therapies, the overall prognosis for GC remains poor. The asymptomatic nature of early-stage disease often leads to delays in diagnosis, with up to 60% of patients presenting with unresectable or metastatic disease at first encounter (3, 4). Common metastatic sites include the liver, peritoneum, lungs, and bone. The presence of peritoneal dissemination in particular is associated with poor response to systemic therapy and a median survival of less than one year (5).

Metastasis to the rectum is an exceedingly rare manifestation of GC and is often under-recognized in clinical practice. Such metastases may present as circumferential rectal wall thickening and mimic primary rectal carcinoma, posing a diagnostic dilemma. Known as Schnitzler’s metastasis, this entity represents peritoneal or submucosal seeding of gastric carcinoma to the rectum or pelvic cavity (6). Signet ring cell carcinoma (SRCC), due to its infiltrative and poorly cohesive nature, is particularly prone to such patterns of dissemination.

In this report, we present a rare case of signet ring cell gastric adenocarcinoma with both peritoneal and rectal metastasis. The patient’s presentation with lower gastrointestinal symptoms and gastric outlet obstruction highlights the diagnostic challenges and underscores the importance of comprehensive histopathologic and endoscopic evaluation in patients with atypical presentations of metastatic GC.

## Case report

**Figure 1:**
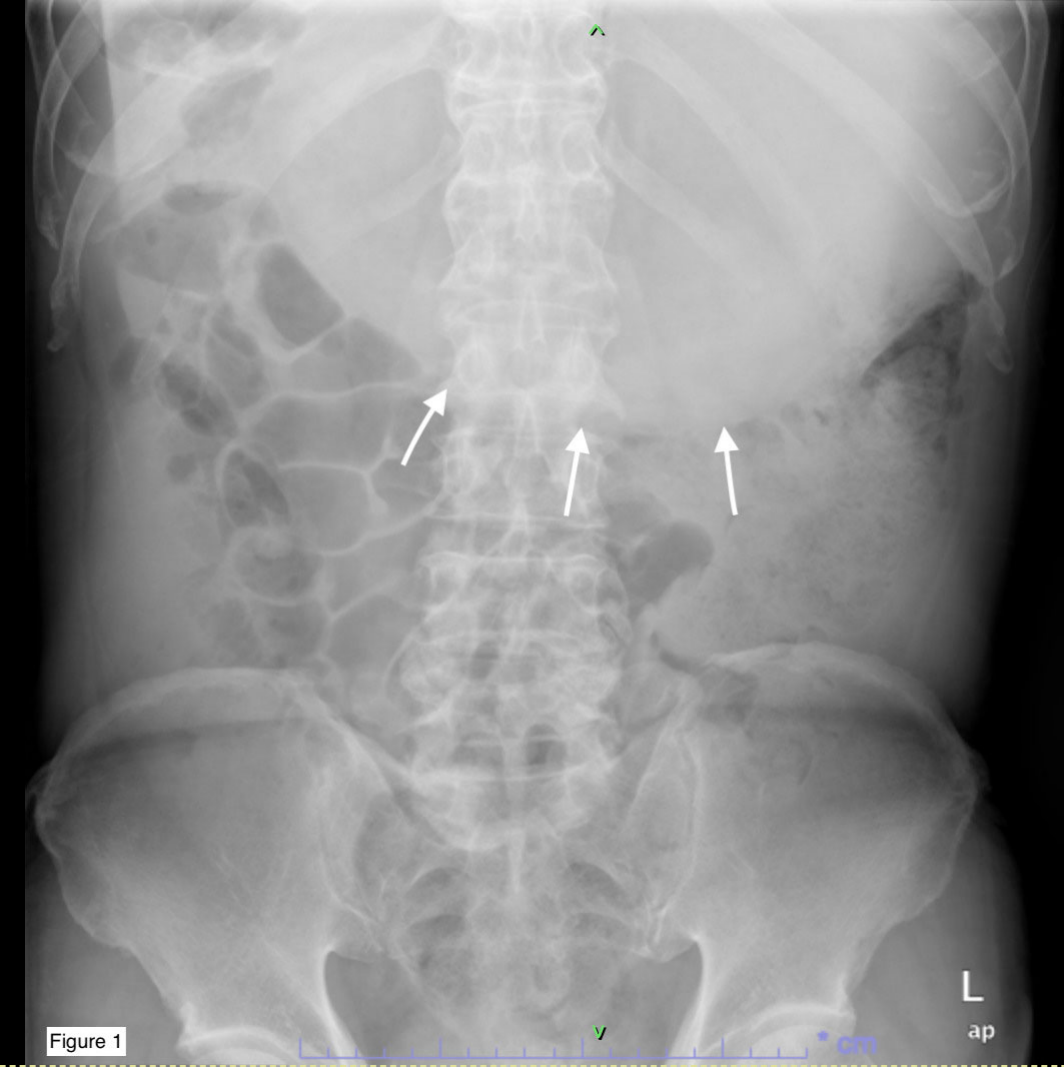
Abdominal X-ray demonstrating gastric distension (white arrows) occupying the upper abdomen, with displacement of adjacent bowel loops.

A 69-year-old male presented to the emergency department with a two-year history of altered bowel habits, constipation, mucous per rectal discharge, and significant constitutional symptoms, including a 10-kg weight loss over six months. Abdominal radiography (**Figure 1**) revealed a right hypochondrial opacity extending across to the left, suggestive of gastric distension, which displaced adjacent bowel loops inferiorly. These findings, coupled with his symptoms, raised concern for gastric outlet obstruction. Abdominal ultrasound confirmed a markedly distended stomach containing heterogeneous intraluminal echogenic contents. Laboratory evaluation showed elevated carbohydrate antigen 19-9 (CA 19-9) at 1064 U/mL (reference range: 0–37 U/mL), with carcinoembryonic antigen (CEA) remaining within normal limits (<1.7 ng/mL). Other routine blood parameters were unremarkable.

**Figure 2:**
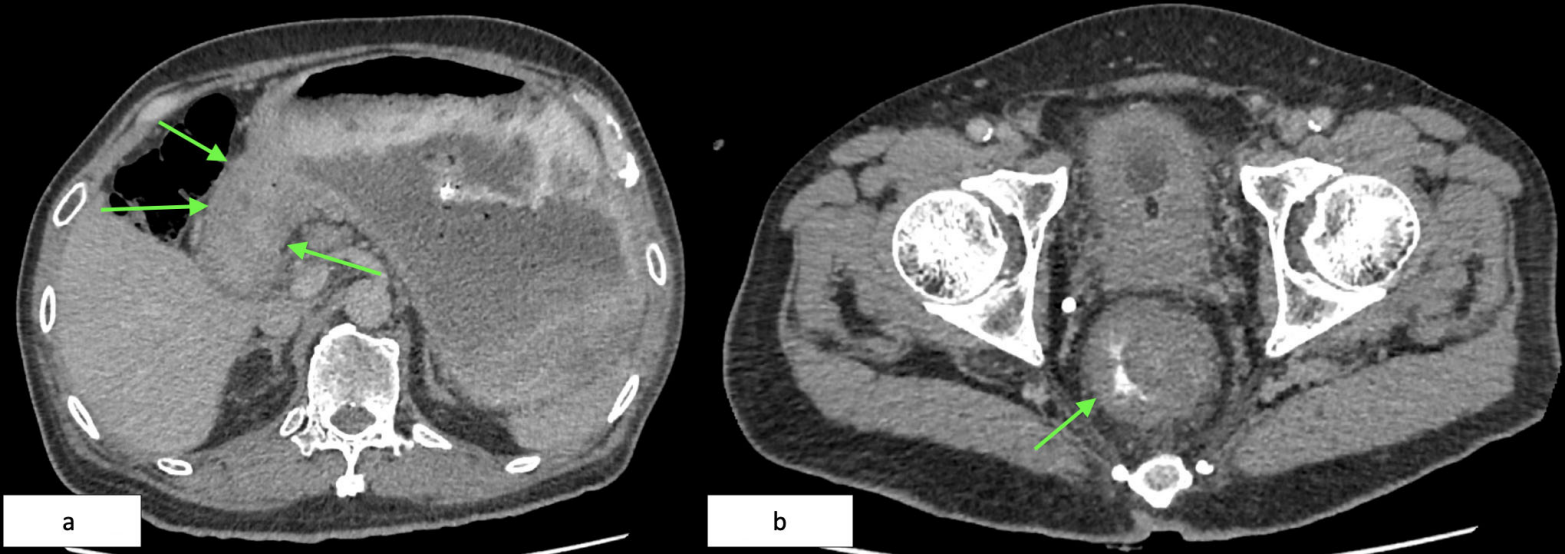
**(a)** Axial CT showing marked thickening of the antrum and pyloric wall (green arrows). **(b)** Axial CECT of the pelvis demonstrating a circumferential rectal soft tissue lesion (green arrow), 9.7 cm in length, starting 2.4 cm from the anal verge.

Contrast-enhanced computed tomography (CT) of the abdomen and pelvis revealed two key findings: (1) circumferential thickening of the gastric antrum extending to the pylorus, and (2) a 9.7 cm long soft tissue mass in the distal rectum, located 2.4 cm from the anal verge (**Figures 2a, b**).

**Figure 3:**
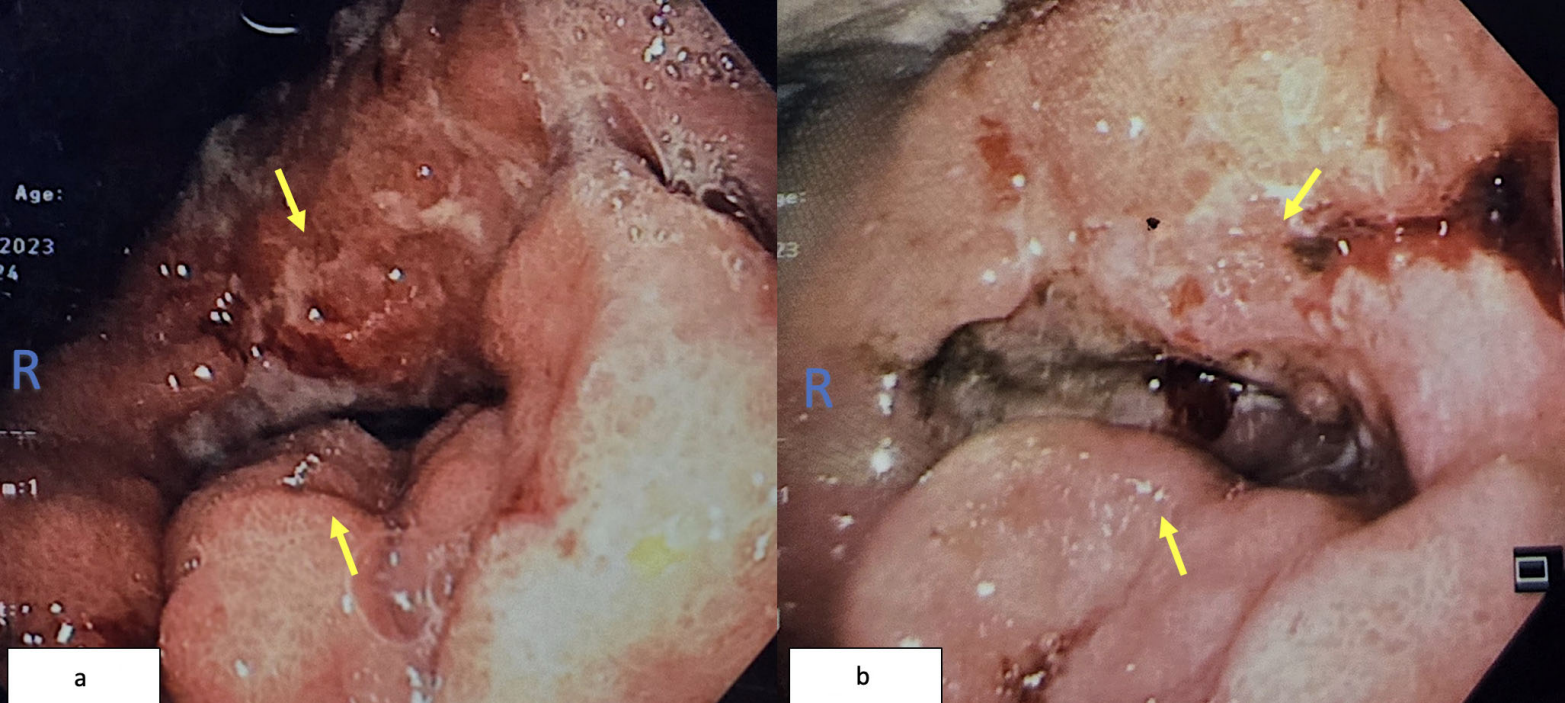
**(a)** Colonoscopy image showing circumferential narrowing due to rectal tumor (yellow arrow). **(b)** OGDS image showing ulcerated antral tumor extending to the pylorus.

**Figure 4:**
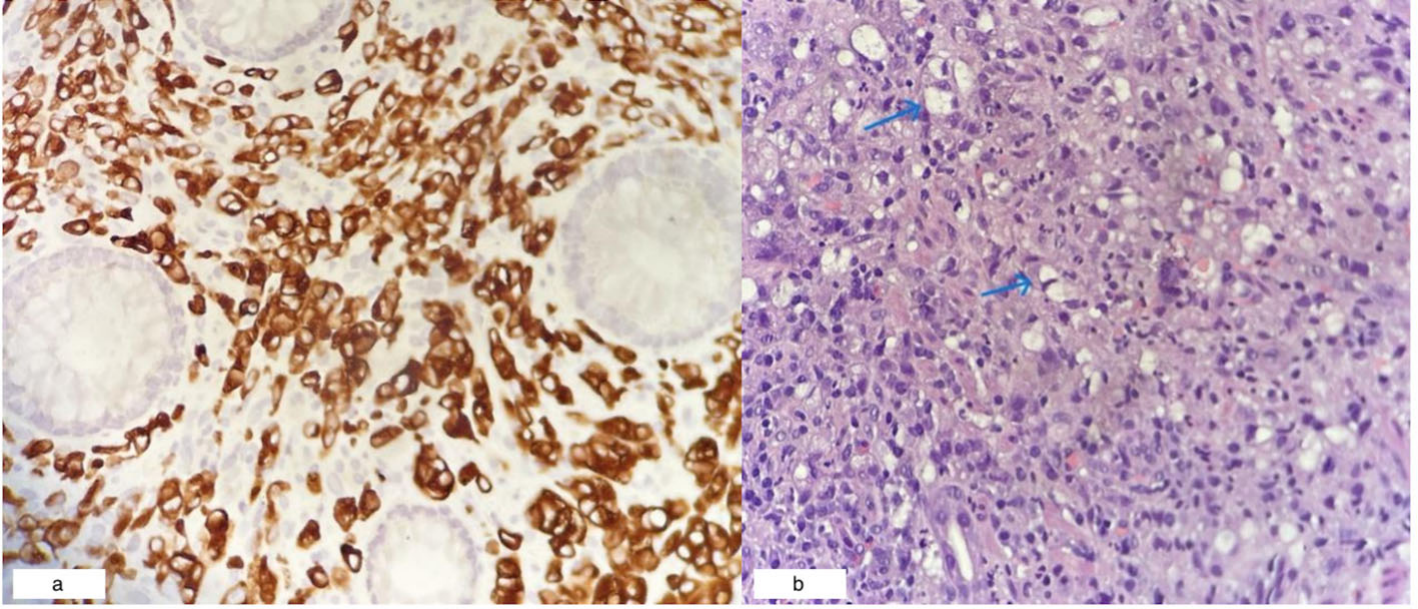
**(a)** Rectal biopsy showing CK7-positive tumor cells (40x magnification). **(b)** Gastric mucosal biopsy revealing signet ring cells (blue arrows; H&E, 40x magnification).

Colonoscopy identified a circumferential rectal lesion approximately 10 cm from the anal verge (**Figure 3a**). An oesophagogastroduodenoscopy (OGDS) revealed a polypoidal, ulcerated lesion extending from the incisura to the pylorus, consistent with a Borrmann type I lesion (**Figure 3b**). Biopsies confirmed poorly cohesive signet ring cell adenocarcinoma of the stomach. Biopsies of the rectal mass showed infiltrative malignant cells positive for cytokeratin AE1/AE3 and CK7, and focally positive for CK20—consistent with metastasis of gastric origin (**Figure 4a**). Histologically, the lamina propria contained dispersed malignant cells, some exhibiting intracytoplasmic mucin vacuoles and signet ring morphology (**Figure 4b**).

Staging laparoscopy revealed multiple peritoneal nodules and puckering of the omentum near the greater curvature. The extensive gastric wall involvement and peritoneal metastases rendered the tumor unresectable. The planned palliative gastrojejunostomy was abandoned intraoperatively due to inability to safely mobilize the stomach. A laparoscopic-assisted transverse colostomy was performed to relieve rectal obstruction.

**Figure 5:**
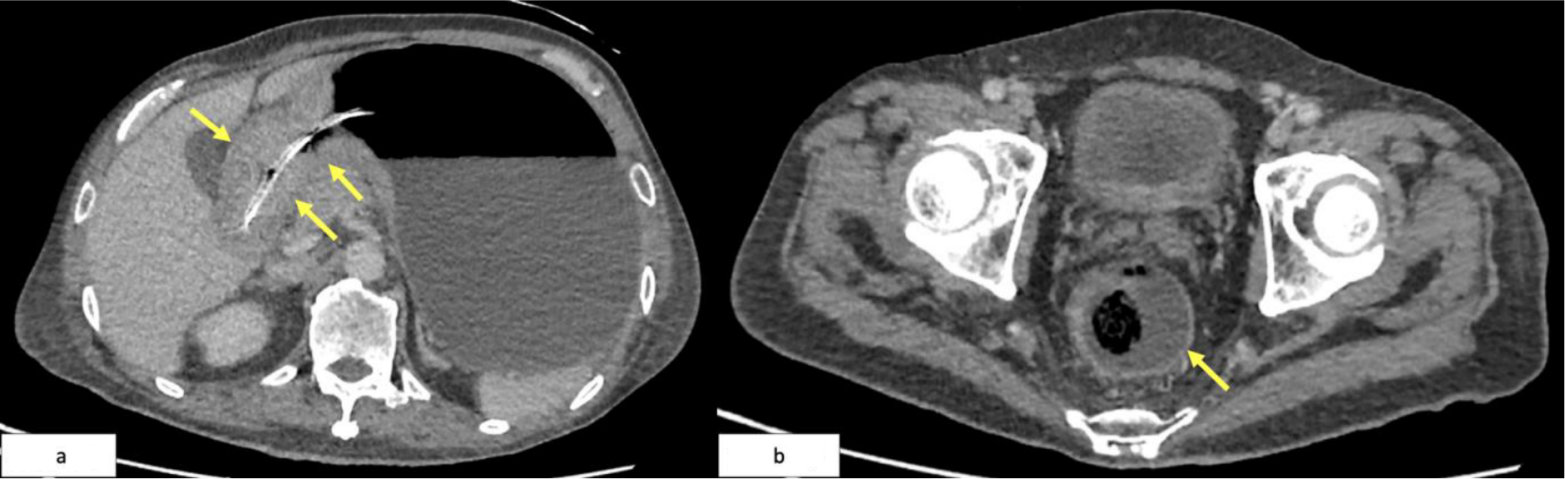
**(a)** Follow-up CT showing interval reduction in antral-pyloric thickening (yellow arrows). **(b)** CT pelvis showing decreased rectal wall thickening (yellow arrow).

The patient commenced systemic palliative chemotherapy, receiving two cycles of FOLFOX (folinic acid, fluorouracil, oxaliplatin), followed by ten cycles of the modified de Gramont (MdG) regimen. Interim CT imaging demonstrated partial radiological response, with reduction in both the gastric wall thickening and rectal tumor burden (**Figures 5a, b**). CEA levels initially decreased from 4.3 to 2.1 ng/mL but rose again to 7.1 ng/mL one month post-chemotherapy.

The patient subsequently re-presented with recurrent gastric outlet obstruction, pleural effusion, and bilateral pulmonary lymphangitic carcinomatosis. Nasojejunal tube repositioning was performed endoscopically. After multidisciplinary consultation and family discussion, a decision was made to transition to best supportive care, including home-based long-term oxygen therapy (LTOT), without further chemotherapy.

## Discussion

Gastric cancer (GC) is a biologically heterogeneous disease that continues to pose major challenges in oncology due to its late-stage diagnosis, poor prognosis, and variable response to treatment. Globally, approximately one-third of patients are diagnosed with metastatic GC at presentation, most frequently involving the liver, peritoneum, lungs, and bone (5). Rectal metastasis, however, remains exceedingly rare and is often underdiagnosed due to its clinical and radiological resemblance to primary rectal carcinoma. This phenomenon, first described by Schnitzler is defined as secondary involvement of the rectum or distal colon by gastric adenocarcinoma, typically via peritoneal or submucosal spread (6).

The rectum may become involved through several metastatic pathways. The most widely accepted mechanism is peritoneal dissemination, where exfoliated tumor cells from the serosal surface of the stomach implant into dependent pelvic structures such as the rectouterine pouch in females or rectovesical pouch in males. These tumor foci often infiltrate the rectal wall submucosally, making them difficult to detect endoscopically until late in the disease course (6).

Other routes include hematogenous spread via the systemic circulation or lymphatic dissemination, especially in cases of diffuse-type gastric adenocarcinomas such as signet ring cell carcinoma (SRCC). SRCC, in particular, is characterized by the loss of E-cadherin and other adhesion molecules, which enhances tumor cell motility, invasiveness, and diffuse peritoneal spread. Molecular studies have shown that SRCC tends to exhibit low HER2 expression, high mucin content, and poor response to chemotherapy contributing to its aggressive nature and atypical metastatic patterns (7).

In our case, the coexistence of peritoneal carcinomatosis and a bulky submucosal rectal mass strongly supports peritoneal seeding as the likely pathway of dissemination. The signet ring morphology observed in both gastric and rectal biopsy specimens further reinforces this metastatic mechanism (7, 8).

One of the most significant clinical challenges in diagnosing gastric SRCC is its nonspecific and often misleading presentation. Early-stage GC may be entirely asymptomatic or present with vague symptoms such as dyspepsia or early satiety. SRCC, in particular, tends to infiltrate the gastric wall diffusely (linitis plastica) without forming a discrete mass, further complicating detection by imaging or endoscopy (9).

In the present case, the patient presented predominantly with lower gastrointestinal symptoms which were altered bowel habits, mucous per rectal discharge, and features of large bowel obstruction, raising suspicion for primary rectal carcinoma. It was only after histological and immunohistochemical analysis that the diagnosis of metastatic gastric cancer was established. The rectal lesion exhibited diffuse infiltration with CK7-positive and focally CK20-positive malignant cells, a pattern that aligns with a gastric rather than colorectal origin.

This underscores the importance of multiple, deep biopsies and the use of a broad immunohistochemical panel in patients with rectal tumors and known upper GI pathology. Failure to distinguish between primary and secondary malignancies can result in significant mismanagement (10).

Janjic et al. (2022) systematically reviewed 62 cases of rectal metastases from gastric cancer and found that the majority were associated with poorly cohesive or signet ring cell histology. Median survival in these patients was generally less than one year, and treatment was largely palliative (7). Tang et al. (2023) also reported similar findings, where patients with rectal metastasis underwent stoma formation, systemic therapy, or palliative measures with limited clinical benefit (3).

Our case is notable in that the patient initially demonstrated partial radiological response to chemotherapy, with reduced gastric and rectal wall thickening and a transient drop in CEA levels. However, this was followed by a rapid increase in CEA and clinical deterioration, highlighting the aggressive and chemoresistant nature of SRCC. Despite receiving FOLFOX and modified de Gramont regimens, the patient’s disease progressed within eight months, ultimately leading to pulmonary lymphangitic carcinomatosis and gastric outlet obstruction.

Systemic chemotherapy remains the mainstay for metastatic GC. However, SRCC shows lower responsiveness compared to intestinal-type adenocarcinoma due to poor drug penetration into diffusely infiltrated tissues and the tumor’s mucinous composition. Targeted therapies such as trastuzumab (for HER2-positive disease) or immunotherapy (PD-L1 inhibitors) have shown promise in selected patients, but were not considered in this case due to negative molecular markers and declining performance status (10).

The role of surgery in metastatic GC is limited to palliative interventions aimed at improving quality of life. In our patient, a laparoscopic-assisted transverse colostomy was performed to relieve rectal obstruction. A planned gastrojejunostomy was intraoperatively abandoned due to extensive tumor infiltration, which precluded safe mobilization of the stomach.

Nutritional support was maintained via a nasojejunal tube, and after disease progression and multidisciplinary discussion, the patient was transitioned to best supportive care, including long-term home oxygen therapy. This decision aligns with the evolving palliative paradigm that prioritizes symptom control, nutrition, and psychosocial support over futile aggressive interventions in end-stage disease (11, 12).

SRCC is not exclusive to the stomach and can originate in other organs, including the colon, ovary (Krukenberg tumor), breast, and gallbladder. Despite differences in anatomical location, SRCC across sites shares common features: diffuse growth, mucin-secreting cytoplasm, and poor cellular cohesion. Studies suggest that cell phenotype, rather than site of origin, may be the dominant factor influencing behavior and prognosis. Nevertheless, immunohistochemistry and clinical context remain critical in distinguishing primary from metastatic disease, particularly in organs like the rectum, where signet ring cells may be seen in both primary and secondary tumors (11, 12).

## Conclusion

Rectal metastasis from gastric adenocarcinoma is an uncommon and diagnostically challenging entity, particularly when presenting with lower gastrointestinal symptoms that mimic primary colorectal malignancies. This case underscores the importance of maintaining a high index of suspicion for secondary rectal lesions in patients with known or suspected gastric cancer, especially those with poorly cohesive or signet ring histology.

Endoscopy with multiple, well-targeted biopsies remains essential for accurate diagnosis, supported by immunohistochemistry to differentiate primary from metastatic lesions. Clinicians should be aware of the aggressive nature of signet ring cell carcinoma and its potential for peritoneal and submucosal spread.

This case also highlights the importance of timely diagnosis and early screening. As recommended by the European Society for Medical Oncology (ESMO) and regional guidelines, high-risk individuals particularly in East Asia should undergo early upper gastrointestinal endoscopy for detection of premalignant or early-stage lesions, which may substantially improve prognosis and expand curative treatment options.

## Data Availability

The raw data supporting the conclusions of this article will be made available by the authors, without undue reservation.

## References

[1] Lim KG Palayan K. A review of gastric cancer research in Malaysia. Asian Pac J Cancer Prev. (2019) 20(1):5–11. doi: 10.31557/APJCP.2019.20.1.5, PMID: 30677863 PMC6485554

[2] Htet H Win TT Wong ST Shamsudin NH Kandasami P. Clinicopathological study of gastric cancer in a Malaysian tertiary public health care centre. Med J Malaysia. (2023) 78(5):616–20., PMID: 37775488

[3] Tang L Li H Lv J Fang C Zhang H Meng J. Rectal metastasis of gastric cancer: a case report. J Int Med Res. (2023) 51:3000605231198407. doi: 10.1177/03000605231198407, PMID: 37815339 PMC10566277

[4] Lordick F Carneiro F Cascinu S Fleitas T Haustermans K Piessen G. Gastric cancer: ESMO Clinical Practice Guideline for diagnosis, treatment and follow-up. Ann Oncol. (2022) 33:1005–20. doi: 10.1016/j.annonc.2022.07.004, PMID: 35914639

[5] Then EO Grantham T Deda X Ramachandran R Gaduputi V. Metastatic gastric cancer to the colon. World J Oncol. (2021) 12:127–31. doi: 10.14740/wjon1375, PMID: 34349858 PMC8297054

[6] Xue H Yang X Shen Q Qu J Qu X Chen Y. Gastric cancer causing Schnitzler's metastasis: case report and systematic review of the features. Acta Oncol. (2025) 64:312–8. doi: 10.2340/1651-226X.2025.41296, PMID: 40008907 PMC11884333

[7] Janjic O Labgaa I Hübner M Demartines N Joliat GR. Metastasis to the rectum: A systematic review of the literature. Eur J Surg Oncol. (2022) 48:822–33. doi: 10.1016/j.ejso.2021.10.004, PMID: 34656391

[8] Mohamed A Nugent K. Colon metastasis from gastric adenocarcinoma: 431. Off J Am Coll Gastroenterol | ACG. (2015) 110:S185. doi: 10.14309/00000434-201510001-00431

[9] Pous A Notario L Hierro C Layos L Bugés C. HER2-positive gastric cancer: the role of immunotherapy and novel therapeutic strategies. Int J Mol Sci. (2023) 24(14):11403. doi: 10.3390/ijms241411403, PMID: 37511163 PMC10380453

[10] Freitas MB Gullo I Leitão D Águas L Oliveira C Polónia A. HER2 and PD-L1 expression in gastric and gastroesophageal junction cancer: insights for combinatorial targeting approaches. Cancers (Basel). (2024) 16(6):1227. doi: 10.3390/cancers16061227, PMID: 38539559 PMC10969487

[11] Takimoto T. Corticosteroids for pulmonary lymphangitic carcinomatosis. BMJ Support Palliat Care. (2024) 13(e3):e951–2. doi: 10.1136/spcare-2022-003983, PMID: 36302614

[12] Ramos MFKP Pereira MA Dias AR Sakamoto E Ribeiro U Jr Zilberstein B. Jejunostomy in the palliative treatment of gastric cancer: A clinical prognostic score. World J Clin Oncol. (2021) 12:935–46. doi: 10.5306/wjco.v12.i10.935, PMID: 34733615 PMC8546652

